# GAR-Net: Guided Attention Residual Network for Polyp Segmentation from Colonoscopy Video Frames

**DOI:** 10.3390/diagnostics13010123

**Published:** 2022-12-30

**Authors:** Joel Raymann, Ratnavel Rajalakshmi

**Affiliations:** 1 Faculty of Mathematics, University of Waterloo, Waterloo, ON N2L 3G1, Canada; jrjasper@uwaterloo.ca; 2 School of Computer Science and Engineering, Vellore Institute of Technology, Chennai 600127, India

**Keywords:** medical image analysis, polyp segmentation, colonoscopy, deep learning, attention mechanism, semantic segmentation, healthcare informatics

## Abstract

Colorectal Cancer is one of the most common cancers found in human beings, and polyps are the predecessor of this cancer. Accurate Computer-Aided polyp detection and segmentation system can help endoscopists to detect abnormal tissues and polyps during colonoscopy examination, thereby reducing the chance of polyps growing into cancer. Many of the existing techniques fail to delineate the polyps accurately and produce a noisy/broken output map if the shape and size of the polyp are irregular or small. We propose an end-to-end pixel-wise polyp segmentation model named Guided Attention Residual Network (GAR-Net) by combining the power of both residual blocks and attention mechanisms to obtain a refined continuous segmentation map. An enhanced Residual Block is proposed that suppresses the noise and captures low-level feature maps, thereby facilitating information flow for a more accurate semantic segmentation. We propose a special learning technique with a novel attention mechanism called Guided Attention Learning that can capture the refined attention maps both in earlier and deeper layers regardless of the size and shape of the polyp. To study the effectiveness of the proposed GAR-Net, various experiments were carried out on two benchmark collections viz., CVC-ClinicDB (CVC-612) and Kvasir-SEG dataset. From the experimental evaluations, it is shown that GAR-Net outperforms other previously proposed models such as FCN8, SegNet, U-Net, U-Net with Gated Attention, ResUNet, and DeepLabv3. Our proposed model achieves 91% Dice co-efficient and 83.12% mean Intersection over Union (mIoU) on the benchmark CVC-ClinicDB (CVC-612) dataset and 89.15% dice co-efficient and 81.58% mean Intersection over Union (mIoU) on the Kvasir-SEG dataset. The proposed GAR-Net model provides a robust solution for polyp segmentation from colonoscopy video frames.

## 1. Introduction

Colorectal Cancer is one of the most common variants of cancer found in human beings. The predecessor of this cancer is the formation of polyps which are found in the colon region. The malignancy degree assessment of colorectal adenocarcinoma consists of two stages. The first stage involves the detection and delineation of polyps from the colon region via colonoscopy examinations. In this stage, the polyps are identified and delineated by an expert clinician. The second stage involves biopsy sample analysis from the segmented polyps using Hematoxylin and Eosin (H&E) staining technique. To reduce the risk of colorectal cancer, the polyps are often analyzed under H&E Staining to assess the malignancy degree and eventually resected [1]. The morphological segmentation of the gland for histopathology is commonly performed by pathologists to determine the stage of cancer/tumor. Accurate segmentation of the glands is a crucial stage in obtaining reliable morphological statistics for quantitative diagnosis. Hence, this is generally performed by the expert pathologist who segments and studies the structure of the glands in the biopsy sample. However, to proceed with the H&E staining on the biopsy sample, there is a need to detect, segment, and delineate polyps from colonoscopy video frames. Unfortunately, even with the careful perusal of each frame in a colonoscopy video, an expert clinician might miss some polyps [2]. Hence, there is a need for a real-time Computer-Aided Detection (CAD) system that can detect and segment polyps in the first stage itself, which is the focus of this research work. The development of such a system can assist clinicians with the delineation of polyps from the colon and can reduce the miss-rate of polyps. 

Automated polyp segmentation is a challenging task mainly due to the varied appearance, shape, and size of polyps. Even though there is a progressive change in the texture, size, and shape of the colorectal polyps in the later stages, it is small and may have no obvious differentiating texture appearance in the earlier stages. This makes it difficult to differentiate with intestinal tissue. Some polyps might even take the entire field of view in the colonoscopy camera. Also, each frame is susceptible to image artifacts, the pattern of shadows, highlights, and even occlusions due to the illuminations in colon screening. In some types of polyps, there is no obvious boundary between a polyp and the surrounding tissue, and the same polyp may look significantly different depending on the camera angle. So, the reliability of polyp segmentation by manual delineation is greatly affected by the lab’s guidelines and the experience of the clinician. Hence, it is hard to determine the gold standard for an automatic segmentation method to deal with all possible types of polyps efficiently, thereby increasing the difficulty of developing a reliable polyp segmenting CAD system. The earlier techniques, such as template matching, contour detections, and texture-based analysis, required manual intervention. Many machine learning and computer vision techniques were applied to solve the polyp segmentation problem. Ref. [3] studied the application of active contours for the segmentation of polyps. As the above approach relies heavily on pre-defined template and shape models, it failed to detect small polyps. Ref. [4] introduced the “depth of valley” concept to detect more general polyp shapes-segmenting the polyps through evaluation of their relationship between the detected edge and the pixels. Their region segmentation algorithm could not handle all types of polyps and lacked robustness. It not only fails to identify some of the small polyps but also segments the endoluminal scene incorrectly as a polyp. To address the challenges in the measurement of segmented colon, different image processing techniques along with statistical analysis were performed [5], but it is a time-consuming and tedious process and could not detect a new type of polyps. Also, the above approaches do not consider contextual information and are not robust.

Recently, deep learning techniques have proven to solve many real-world problems with high robustness. Many variants of deep neural network architectures have been reported in the literature for the task of semantic segmentation for various applications, viz., remotely sensed data segmentation [6], road-scene segmentation, indoor scene segmentation [7], and biomedical image segmentation [8]. The study by [9] shows the superior performance of a Fully Convolutional Network (FCN) for semantic segmentation in colonoscopy images but is not able to yield an accurate prediction in the case of noisy images. In another work by [10], FCNs are employed for the segmentation of polyps along with a probabilistic-based post-processing algorithm. In this approach, a heuristic-based threshold was used to differentiate the polyp from the normal tissues, which is error-prone and could not characterize well all types of polyps that are irregular in shape and size. 

The incorporation of the attention mechanism in deep neural networks can help the model generate a less noisy and more refined output map. For the diagnosis of coronary artery diseases [11], the attention-based vessel segmentation approach has been applied by adding low-level and high-level features. The sparse contour attention mechanism has been applied to obtain accurate region boundaries for liver segmentation in abdominal CT images [12]. They combined the sparse contour attention along with an auto-context algorithm and applied the self-supervised algorithm to improve the performance of segmentation.

In this research work, we propose a novel deep end-to-end architecture for segmenting polyps from colon screening frames by employing a modified residual network with a special attention mechanism. In the proposed approach, the lower semantic information captured in initial layers is also considered to handle different sizes, and shapes of polyps and to suppress the noise in the input. A novel Guided Attention mechanism is proposed that allows the model to generate and apply attention maps for each feature map in the input to obtain a refined and accurate segmentation output. We evaluated our model on two datasets, viz., the CVC-ClinicDB polyp dataset and the recent Kvasir-Seg [13] dataset and achieved state-of-the-art performance over other proposed deep learning models. Our significant contributions to this research work are summarized below:

- A novel end-to-end deep learning framework for segmenting polyps from colonoscopy video frames.
- A modified and enhanced Residual Block is proposed that suppresses the noise and preserves the low-level feature maps for a more accurate semantic segmentation.
- A special learning technique is introduced with a novel attention mechanism for obtaining an accurate segmentation map.
- A novel attention mechanism to capture the refined attention maps regardless of the size and shape of the polyp, also under improper illumination conditions.
- Design of a competitive and robust model with consistent performance over the benchmark CVC-ClinicDB dataset and the Kvasir-SEG Dataset.

This paper is organized as follows: The related works are discussed in Section 2. The proposed methodology is elaborated on in Section 3. The results and discussions of all experiments are presented in Section 4 followed by a conclusion in Section 5. 

## 2. Related Works

Many machine learning and deep learning methods were proposed by various researchers in the field of medical image analysis that includes, including lesion identification in pulmonary nodules, lung nodules, colonial cancer, brain lesion segmentation, polyp segmentation, etc. Ref. [14] presented a detailed survey on image-based cancer detection using various deep learning architectures. In their study, they have outlined the methods suitable for different types of cancers, including breast cancer, lung cancer, skin cancer, prostate cancer, brain cancer, colonial cancer, cervical cancer, bladder cancer, etc. They discussed the issues of the lack of large data sets required for training the better models and the various available solutions like image augmentation and transfer learning to address the same. Computer Aided Detection (CAD) plays a major role in detecting lesions by providing assistance in the workflow of the radiologist. Ref. [15] studied the problems in identifying lesions in pulmonary nodules. To address the issues of highly imbalanced data and to reduce the false negatives in classification, they have proposed a multi-kernel approach. In their work, feature fusion and oversampling have been employed to select the important subset of relevant features. Ref. [16] proposed a deep learning-based technique for lung nodule detection on low-dose thoracic helical CT (LDCT) dataset and exploited the Convolutional Neural Network (CNN) and the traditional Artificial Neural Network (ANN). In their study, they observed that CNN architecture is good at capturing low-level and high-level features compared to ANN. 

In this research work, we mainly focus on the polyp segmentation problem. Many solutions were proposed to automate the segmentation process of polyps in colon screening. In many cases, polyps have well-defined shapes and structures. Hence, earlier methods tried to leverage this to perform polyp segmentation. Ref. [17] proposed the usage of the canny edge detector technique to process the images and identify relevant edges with the assistance of template matching techniques. Following this, Ref. [3] studied the application of active contours for the segmentation of polyps, but these template-based models are not suitable for detecting small polyps. 

Many texture-based methods were also introduced as a solution to the polyp segmentation problem. Karkanis et al. [18] used Grey-Level Co-occurrence Matrix (GLCM) and wavelet methods to detect polyps. Ref. [19] proposed an SVM-based method to detect and classify the abnormalities in endoscopic images. They mainly focused on feature extraction and developed an algorithm that can assign the weights for the relevant features and to remove the useless ones from the hand-crafted features that were extracted from the endoscopic image. With their deep sparse SVM-based approach, they were able to reduce the feature dimension and build a better model for classifying the endoscopic images on their own dataset. In another work, an SVM-based approach with hand-crafted features was applied [19] to detect the abnormalities in endoscopic images. As their SVM model could not handle the noisy and poor-quality images, they introduced a rejection stage. The image quality was pre-assessed based on its pixels, and only if it was at an acceptable level was it fed to the next segmentation stage by SVM. Otherwise, the image was rejected in the pre-processing stage itself, thereby limiting its usage. Ref. [20] attempted to characterize the polyps by traditional methods such as edge detection, feature extraction, and feature reduction and then applying an ensemble-based approach for classification. But these handcrafted features were not accurate for delineating the polyp boundary.

For colonic polyp measurement, Ref. [21] followed a topographical height map approach. They computed the topographic features from the generated height maps of the polyp. The concentric patterns from the height maps were then used for the texture analysis. By applying the SVM classifier, the normal surface of the colon was differentiated from the colonic polyps. They analyzed the experimental measurements with that of the height map approach, and it was found to be more efficient than the other methods. Recently, a rapid change has been observed in these tasks as Convolutional Neural Networks (CNN) are being employed to provide more robustness compared to hand-crafted features.

All the above approaches are not sufficient for the polyp segmentation task, as they fail to capture the contextual information from the images. To address the above problems, Fully Convolutional Networks (FCN) proposed by [22] were adapted for semantic segmentation. Later, U-Net [8] architecture was widely used for developing an end-to-end model for semantic segmentation. Following these works, Ref. [9] proposed a standard FCN for the segmentation of polyps and used the Random Forest algorithm to decrease the false positive results. 

From neural machine translation to sentence classification, applying the attention mechanism has allowed models to focus on important features, resulting in less noisy, more refined feature maps [23]. Recently, Ref. [24] tried to incorporate an attention mechanism, and their study suggests that attention mechanisms can substantially reduce the noise in the output and help the model generate a more refined output map. Hence, it can be concluded that a better attention mechanism can further improve the performance of the models. Ref. [25] tried various methods, from Machine Learning to Deep CNN models, and suggested various methods for classifying Gastrointestinal (GI) tract diseases. 

As deep learning methods have proven to learn robust features for segmentation problems, we have applied them to get a robust model for the segmentation of polyps of varied textures, shapes, and sizes. However, recent deep learning approaches in polyp segmentation output noisy outputs and broken segmentation maps. Hence, the incorporation of a better attention mechanism can help the model generate a less noisy and more refined output map. In this work, we have proposed a Guided Attention Residual Network (GAR-Net) by employing both residual blocks and attention mechanisms to obtain a refined segmentation map for polyp segmentation.

## 3. Proposed Methodology

The proposed Guided Attention Residual Network (GAR-Net) architecture for polyp segmentation is presented here with its building blocks. We present our novel Guided Attention Learning (GAL) along with the proposed loss function of the proposed GAR-Net model.

### 3.1. Building Blocks of GAR-Net

The basic residual block, which is incorporated in the proposed model, is presented below. It is followed by our modified residual block and the Guided Attention Module (GAM)-the attention mechanism that we have proposed in this research. 

#### 3.1.1. Residual Block

**Figure 1:**
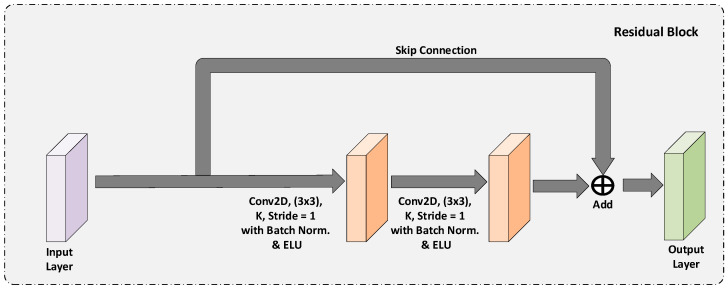
The overview of the Residual Block used.

The residual block, ResNet [26], provides an exceptional performance boost and faster convergence because of the skip connections present in the block. The authors have shown that, even if the network depth increases by stacking many layers, it is not guaranteed to get an increase in accuracy. Because the network depth is limited by the issue of vanishing gradients. The gradient becomes very small, or even zero, in the earlier layers, and in such cases, even the shallow networks perform better than the deeper networks. To address this issue, the concept of skip connections was introduced, which provides better gradient flow and helps in avoiding exploding and vanishing gradients. By using skip connections, the information captured in the initial layers can be allowed to the later layers, thereby enabling the later layers to learn the lower semantic information captured in earlier layers. It is incorporated in many deep learning models to improve the performance of classification and semantic segmentation problems [6]. Figure 1 shows the overview of the used residual block. 

The K in Figure 1 is the number of filters applied in the convolution operation used in the residual block. Although residual networks have shown great performance, they have a drawback of noise added to the low-level features captured in the earlier stages of the network due to the plain addition of the skip connection. The ResNet adds the skip connection bypassing the non-linear transformations with an identify function, which helps the gradient to flow directly to the earlier layers from later layers. Even though it preserves the information from earlier layers by additive identity transformation, noise is also added. To suppress that noise, we have modified this architecture to learn the refined low-level feature maps by adding a convolutional block in the skip connection, as explained below.

#### 3.1.2. Modified Residual Block

**Figure 2:**
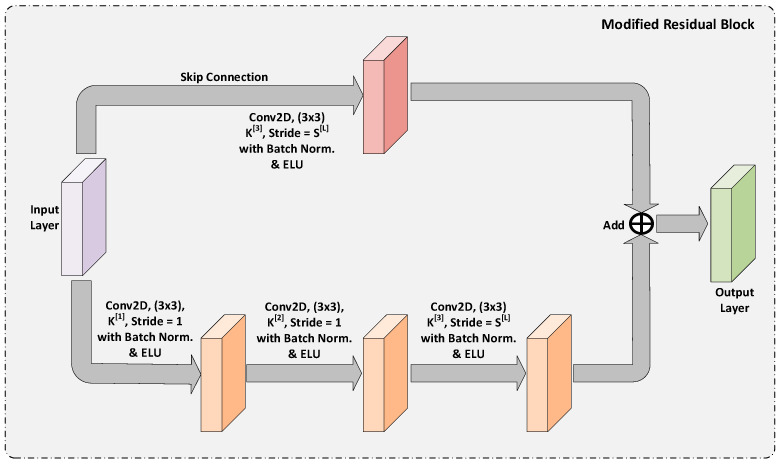
The overview of the Modified Residual Block.

We proposed a modified residual block and applied it in our GAR-Net model to alleviate the problem found in normal residual networks. We have introduced a convolution layer in the skip connection before the addition, and this helps the model in two ways, (i) suppresses the amount of noise added in the feature maps and (ii) helps the model to learn in the skip connection and to capture refined low-level feature maps. The overview of the modified residual block is shown in Figure 2.

In the proposed modified residual block, the two important parameters are $K^{\left[i\right]}$ and $S^{\left[L\right]}$ . $K^{\left[i\right]}$ is the number of filters applied at the ith convolution operation in the main flow. $S^{\left[L\right]}$ indicates the striding for the last convolution layer. The input layer is taken through two paths-main paths and a skip path. In the main path, a series of convolutions are applied, and the last convolution alone with a stride of $S^{\left[L\right]}$ . In the skip connection, a single convolution is introduced with a stride of $S^{\left[L\right]}$ . Finally, the output of both paths is added to get the final output feature maps.

#### 3.1.3. Guided Attention Module

**Figure 3:**
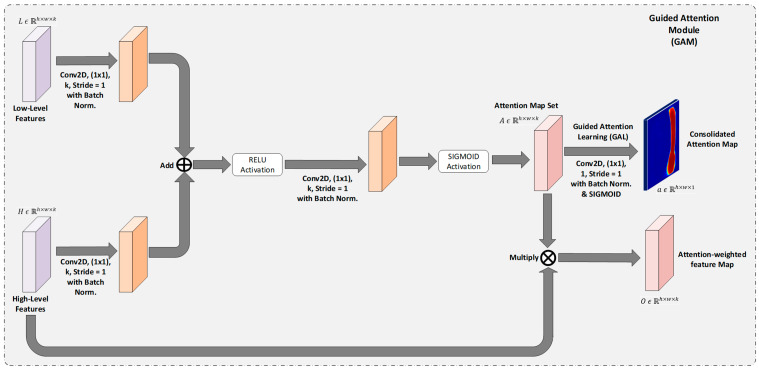
The overview of the Guided Attention Module with Guided Attention Learning.

Attention mechanisms can drastically improve the performance of deep learning models. A general attention mechanism consists of a query(Q) and a value(V) and it boosts the set of features present in the values to which the query is related. In other words, the attention lets the model focus on the information it needs from the values using the query. Ref. [24] proposed an attention-gated mechanism for semantic segmentation tasks on medical images. The major drawback of this work is that a single attention map is broadcasted and multiplied across the entire set of feature maps. This can lead to a noisy feature map at the beginning of the training making the entire training process slower with a higher convergence time. Furthermore, this can disrupt the feature map set, as each feature map consists of information that facilitates the model to output more refined output. To address the above drawbacks, we propose a new attention mechanism called Guided Attention Module (GAM), which allows the model to generate and apply attention maps for each feature map in the input feature sets. Furthermore, GAM is fine-tuned to generate a more refined attention map with a special technique called Guided Attention Learning (GAL). The overview of the proposed Guided Attention Module is presented in Figure 3.

Consider a low-level feature map $L\;\epsilon \;{\mathbb{R}}^{h\times w\times k}$ and a high-level feature map $H\;\epsilon \;{\mathbb{R}}^{h\times w\times k}$ , where h, w, k denote the height, width and number of channels in the feature map set. To apply GAM, a 1×1 convolution with k filters is applied on the low-level feature map L and another 1 × 1 convolution with k filters is applied on high-level feature map H , simultaneously. The outputs of these two operations are added together and activated with a non-linear relu() function. This output is passed through a 1×1 convolution with k filters and is activated with a non-linear sigmoid() function to obtain the attention map set $A\;\epsilon \;{\mathbb{R}}^{h\times w\times k}$ . A is then multiplied elementwise with the up-sampled low-level features to get the final attention-weighted feature maps $O\;\epsilon \;{\mathbb{R}}^{h\times w\times k}$ . Batch-Normalization [27] is applied before every non-linear activation function to improve the training process. With the attention map set A , we obtain individual pixel-wise attention maps for each feature map, thereby allowing the noise due to multiplying a single attention map. To further refine the attention maps, we train this set of attention maps A using our proposed Guided Attention Learning (GAL) technique.

### 3.2. GAR-Net for Polyp Segmentation

The working principle of the proposed Guided Attention Learning (GAL), along with the loss function used in our GAR-Net architecture, is presented below.

#### 3.2.1. GAR-Net Architecture

**Figure 4:**
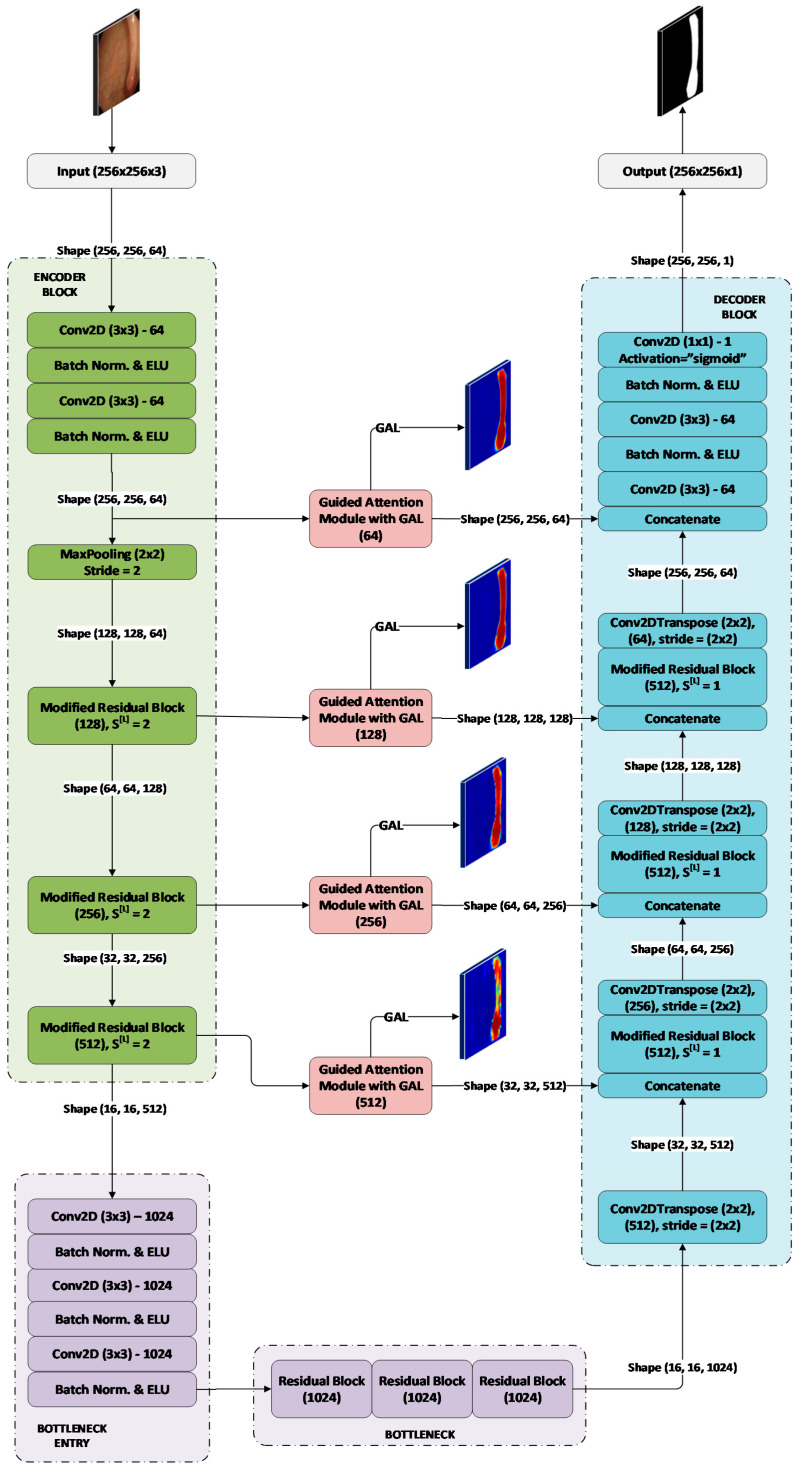
The overview of the proposed GAR-Net Architecture.

The overview of GAR-Net architecture is shown in Figure 4. The input of the proposed GAR-Net architecture is 256×256×3 normalized between [0.0, 1.0]. The encoder learns the features from the given input, which is then fed to a set of bottleneck layers to get the consolidated encoded feature maps. The decoder reconstructs the output from the encoded feature maps. The skip connection plays a crucial step in the reconstruction process as it helps the decoder access the low-level information from the encoder layers. These low-level feature maps consist of the spatial/location information and help the decoder reconstruct the output map, viz., the skip connection. We incorporated a Guided Attention Module (GAM) with GAL to effectively reconstruct a refined output map at each stage in the decoder. The GAM takes the up-sampled High-Level feature maps and the low-level feature maps from the skip connection and merges the map efficiently to reconstruct a refined output map in the decoder.

#### 3.2.2. Guided Attention Learning

The high-level features consist of more information pertinent to the segmentation task, while the low-level features consist of information that helps the model reconstruct the output segmentation map. The purpose of attention is to use the information from the high-level coarse feature map, and spatial/location information from the low-level feature maps to reconstruct a more refined set of feature maps, hence, combining both high-level and low-level feature maps effectively. However, an attention mechanism can be detrimental to the performance of the model if it fails to capture meaningful information from the training set. To address this issue, we propose Guided Attention Learning (GAL) mechanism. Guided Attention Learning is a training mechanism that allows one to train the attention map according to their needs and to have control over its focus on specific features. To implement GAL, a simple 1×1 convolution is applied with a single filter over the attention map set A . It is then followed by batch normalization and a non-linear sigmoid() activation. GAL results in a consolidated single attention map $a\;\epsilon \;{\mathbb{R}}^{height\times width\times 1}$ representing all the information present in the attention map set A . Next, we train the output a using binary cross-entropy to suit our desired attention map. The incorporation of GAL in the loss function for our proposed GAR-Net is elaborated below.

#### 3.2.3. Loss Function for GAR-Net

The model proposed in Figure 4 gives five outputs, namely: The main segmentation map output ( $y_{pred}$ ), and the attention maps ( $a_{1},a_{2},\;a_{3},\;a_{4}$ ) from the GAM module. For training the model, the dice loss $L_{main}$ for our main output ( $y_{pred}$ ) is initially calculated using the following equation:

(1) $$\begin{array}{c} L_{main}=1.0-\frac{2\;\left|\;y_{pred}\;\cap \;y_{gnd\;}\right|\;}{\left|y_{pred}\right|+\left|y_{gnd}\right|}\; \end{array}$$

where, $y_{pred}$ is the set of pixels in the predicted output map, $y_{gnd}$ is the set of pixels in the ground truth segmentation map. 

In general, the attention layer is allowed to train on its own. But in this proposed work, Guided Attention Learning is introduced to control what must be focused on by the attention mechanism. The crux of GAL is to train the attention mechanism on where to focus. To focus on the region of interest using the proposed attention mechanism (GAM), each attention layer is trained alongside the main objective in a multi-tasked learning approach. For each attention map $a_{i}\;\epsilon \;\left[a_{1},\;a_{2},\;a_{3},\;a_{4}\right]$ , the binary cross-entropy loss is calculated. At this stage, the following question arises in our mind: With respect to what will we find the loss? What is the ground truth attention map? This can be answered by understanding what a perfect attention map is. The main reason for applying attention at each up-sampling stage is that we wish to reconstruct higher resolution feature maps effectively using the up-sampled high-level features maps and the low-level skip connection feature maps. A perfect attention map, in such cases, must resemble the segmentation map. Hence, the ground truth for the attention maps will simply be the segmentation map itself but resized to the size of that attention map $a_{i}$ . For example, let’s take $a_{3}\;\epsilon \;{\mathbb{R}}^{64\times 64}$ and $y_{gnd}\;\epsilon \;{\mathbb{R}}^{256\times 256}$ be the ground-truth segmentation map, then the ground truth attention map for $a_{3}$ will be $y_{gnd}$ itself but resized to 64×64 to match the dimension of $a_{3}$ . By using this method, we can train all four attention mechanisms using binary cross-entropy. The guided attention loss $L_{a_{i}}$ for each attention map $a_{i}\;\epsilon \;\left[a_{1},\;a_{2},\;a_{3},\;a_{4}\right]$ can be calculated by the following equation:

(2) $$\begin{array}{c} L_{a}=-\underset{x\;\epsilon \;\Omega }{\sum }\log\left(P\left(a_{ipred}\left(x\right),\;a_{ignd}\left(x\right)\right)\right)\; \end{array}$$

where Ω implies the image space from which each pixel x is considered. $P\left(a_{i_{pred}}\left(x\right),\;a_{i_{gnd}}\left(x\right)\right)$ is the calculated sigmoid probability of the attention map $a_{i}$ on the corresponding pixel x . The total loss $L_{total}$ is the aggregation of all these loss functions and can be calculated by the following equation:

(3) $$\begin{array}{c} L_{total}=L_{main}+\overset{4}{\underset{i=1}{\sum }}L_{a_{i}}\; \end{array}$$

## 4. Results and Discussion:

To evaluate the performance of the proposed method, various experiments were carried out. A detailed discussion of the experimental setup and results are presented in this section, along with the attention visualization.

### 4.1. Dataset

For the polyp segmentation task from images, each pixel in the training images must be labeled as either the polyp class or the non-polyp class. For evaluating our proposed GAR-Net model, we have used two benchmark datasets-the CVC-ClinicDB [28] and the Kvasir-SEG [13]. The CVC-ClinicDB is an open-access dataset of 612 images, each with a resolution of 384×288 , acquired from 31 colonoscopy sequences. The Kvasir-SEG dataset is a considerably huge dataset of 1000 polyp images given along with their annotated ground truth masks. The annotations of these masks were performed by expert endoscopists from the Oslo University Hospital (Norway). The resolution of the individual images in the Kvasir-SEG dataset varies from 300×300 to 1920×1080 and beyond in certain cases.

### 4.2. Experimental Setup

All the architectures used for comparison and evaluation in this research were implemented in TensorFlow version 2.0. The models were built using the high-level Keras API, while the training process was performed with TensorFlow 2.0’s gradient tape training mechanism. The loss and metrics were evaluated using TensorLayer [29]. The models were trained with an Intel i7-7700HQ CPU with 16GB DDR4-2400MHz RAM and an Nvidia GTX 1060 GPU with 6GB VRAM. The system environment is Windows 10 OS and had the latest Nvidia CUDA v10.2 installed with CuDNN v7.6.5. The implementation code is developed in python version 3.6.8.

### 4.3. Training Setup

The size of the image within the same dataset varies significantly. Hence, for effective GPU utilization and to reduce the training time, we resized the training images to 270×270 and applied a series of augmentation techniques to increase the training dataset. The final input training images of size 256×256 are acquired from random cropping of the augmented training images. We extensively used data augmentation techniques such as random HSV saturation, random brightness, random contrast, random horizontal and vertical flip, random scaling, and random rotation (rotation range $\epsilon \;\left[0^{o},\;90^{o}\right]$ ). We also used noise and blurring augmentation techniques such as median blur, gaussian blur, motion blur, and gaussian noise. It is to be noted that augmentation techniques were applied only during the training process and not for testing and validation. For validation and testing, we resized the input image to 256×256 using a bicubic resizing algorithm [30] before feeding the images to the model.

For all the experiments, 80% of the dataset was used for training, 10% for validation, and the remaining 10% for testing. All the models were trained for 120 epochs with a batch size of 4. We used Adam optimizer and Dice loss to train the model. The initial learning rate was set to $1.0\times 10^{-4}$ and was eventually reduced upon reaching a plateau in the validation loss. This type of learning rate reduction is called learning rate plateau-a technique that monitors the validation loss and reduces the learning rate upon no improvement in the validation loss for the corresponding epoch. For testing and evaluating the model after training, we used the models which gave the least validation loss. All hyper-parameters were tuned manually and found that the above setting helped all the models perform well on both datasets. 

### 4.4. Results

We implemented and compared the proposed model with the existing techniques to study the effectiveness of this approach. We compared our proposed GAR-Net model with FCN [22], U-Net [8], U-Net with gated attention [24], SegNet [7], ResUNet [6], and DeepLabv3 [31]. We implemented all the aforementioned models on our own except the DeepLabv3 model. The implementation code for the DeepLabv3 model was taken from their GitHub repository [*GitHubRepo*]. We trained all the models on both the CVC-ClinicDB dataset and the Kvasir-SEG dataset. We used the dice coefficient and meant Intersection over Union (mIoU) as our main metric of evaluation, as these metrics are much more suitable for evaluating semantic segmentation tasks. 

#### 4.4.1. Results on CVC-ClinicDB Dataset

The models were evaluated with the CVC-ClinicDB dataset, and the results are shown in Table 1. 

From Table 1, our proposed model outperforms other models with the highest dice coefficient (0.910) and mIoU score (0.831). Even in the pixel-wise accuracy (0.983), the proposed model outperforms all the other models.

**Figure 5:**
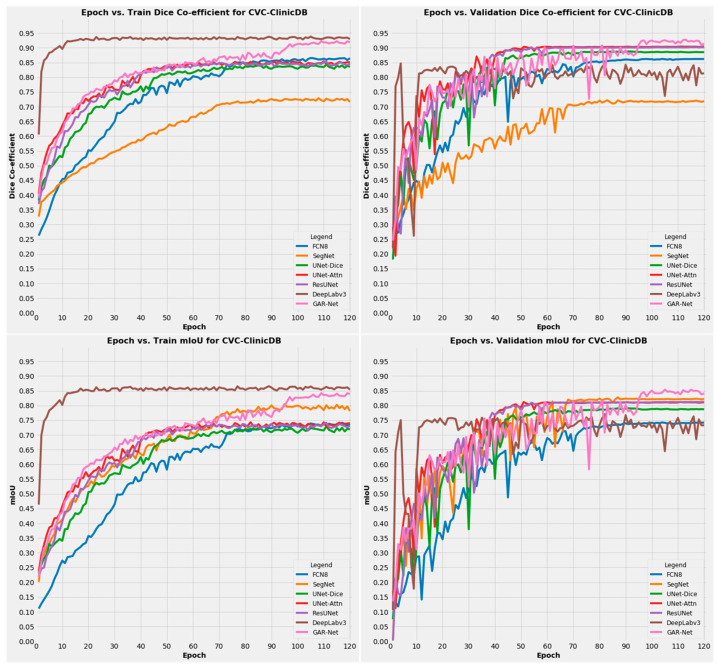
Training and Validation results on the CVC-ClinicDB dataset.

The performance of the proposed GAR-Net was analyzed by plotting a graph with the obtained Dice Coefficient and mIoU for the training set (left side of the graph) and validation set (right side of the graph), respectively. From Figure 5, it is observed that DeepLabv3 performs well only on the training set (depicted in the top-left and bottom-left of the graph) but lacks generalization, which has led to poor performance on the validation set (top-right and bottom-right). But our proposed model GAR-Net shows its superiority in the validation set also with a competitive performance in the training set, hence, providing a robust solution on the CVC-ClinicDB dataset. Similarly, other existing models also fail to provide a robust solution, and the performance on the validation set is not as good as the proposed GAR-Net. So, we can conclude that our proposed model provides consistent performance in terms of Dice Co-efficient and mIoU over both the training and validation set.

#### 4.4.2. Results on Kvasir-SEG Dataset

All the models were also evaluated on the Kvasir-SEG dataset, and the results are summarized in Table 2. 

From the results, it is evident that our proposed model outperforms all the other models with the highest dice coefficient (0.891), mIoU score (0.815), and pixel-wise accuracy (0.971). 

**Figure 6:**
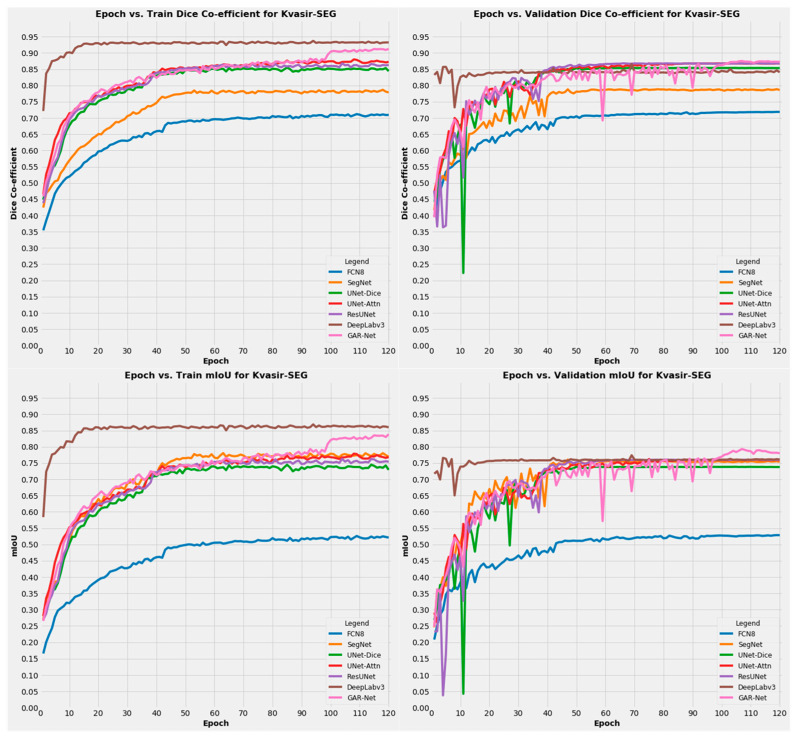
The Training and Validation Graphs for the Kvasir-SEG Dataset training.

From the plotted graphs, as shown in Figure 6, we can observe that our model performance is consistent in both the training and validation set on the Kvasir-SEG dataset also. Our proposed model shows competitive performance in terms of dice coefficient and outperforms all other models in mIoU in the validation set, thus providing a robust solution. 

### 4.5. Further Discussions

To evaluate the significance of the proposed Guided Attention mechanism, we present the visualization of the outputs of all the models for some test samples randomly taken from both datasets and compare its performance with existing methods

#### 4.5.1. Output Visualization for CVC-ClinicDB Dataset

**Figure 7:**
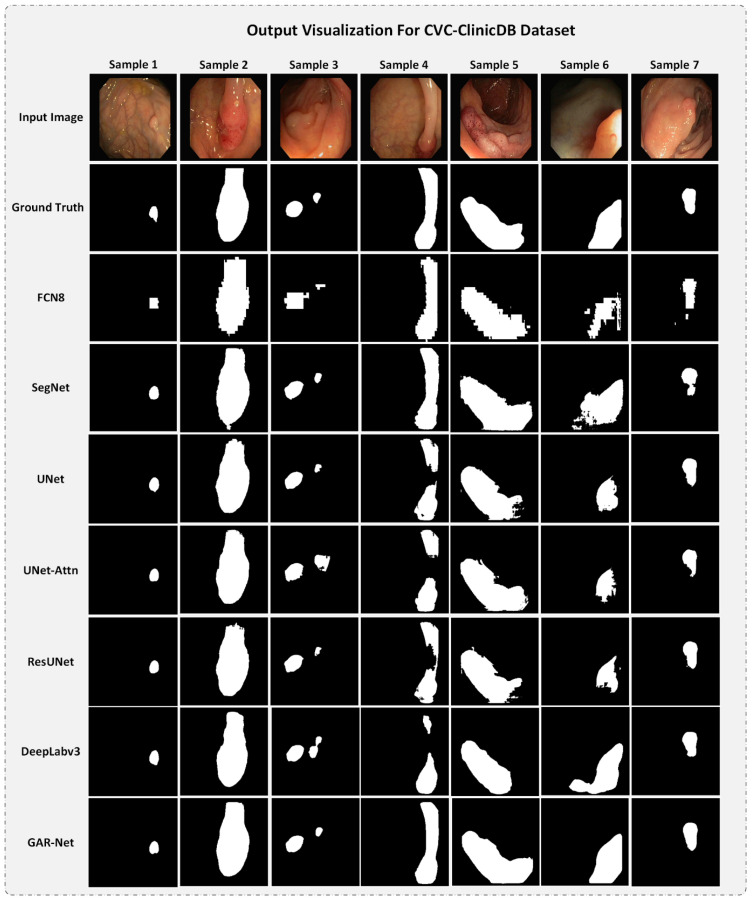
The output comparison for all the models on the CVC-ClinicDB Dataset.

A set of random test samples were taken from the CVC-ClinicDB dataset and is visualized. The comparison with all the other trained models is depicted in Figure 7. 

From Figure 7, it can be observed that our proposed model produces a more refined map than all other models. It is also evident that almost all models predict well for samples 1 and 2. In sample 3, DeepLabv3 predicts with noise, while in sample 4 and sample 5, the DeepLabv3 model fails to provide proper continuous maps. All models, except our proposed GAR-Net model, fail to capture a refined output map for the Sample 6 model. This is due to improper illumination. However, our proposed model adapts to it to an extent and provides an overall refined output map. Hence, we can conclude that our model outperforms all the other models showing the superiority of the proposed GAR-Net architecture on the CVC-ClinicDB dataset.

#### 4.5.2. Output Visualization for Kvasir-SEG dataset

**Figure 8:**
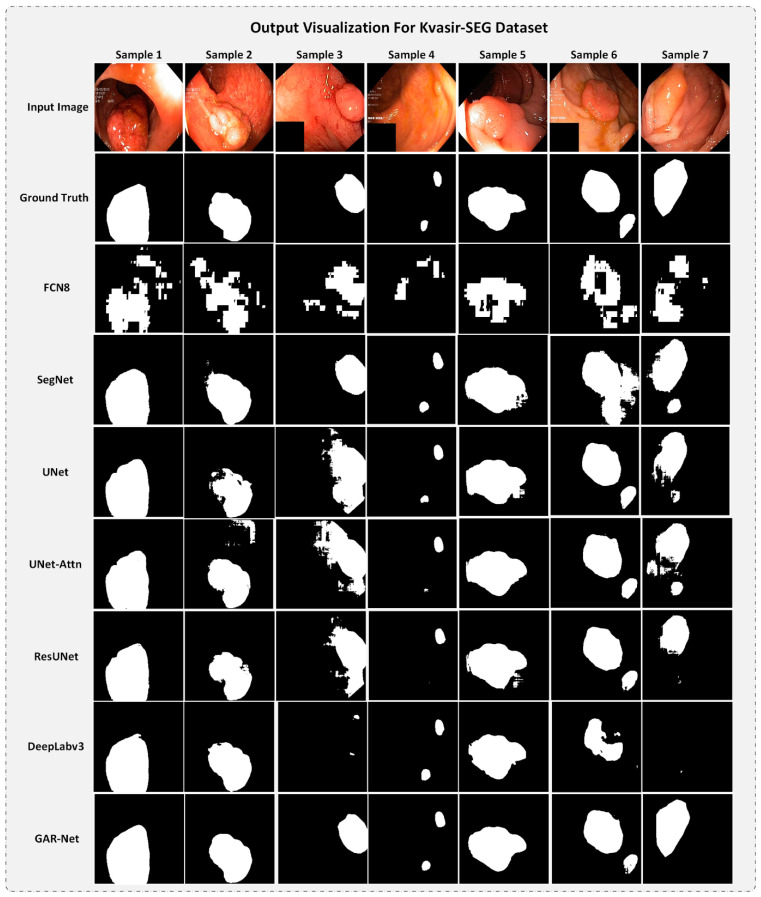
The output comparison for all the models on the Kvasir-SEG Dataset.

A set of random test samples were taken from the Kvasir-SEG dataset and visualized (Figure 8) and compared with all the trained models. 

From Figure 8, one can quickly notice the visual noises present in the Kvasir-SEG input images. These noises include writings, random color patches, etc., which make the Kvasir-SEG dataset, a challenging dataset. FCN8 fails to provide any refined output for all the samples. Most models perform well on sample 1, but UNet and UNet-Attention fail to handle the visual noises present in the input image of samples 2, 3, and 7, hence, resulting in a broken and irregular output map. SegNet provides a decent output compared to UNet and UNet-Attention on all the samples except sample 6, where it fails to find two separate polyp objects. DeepLabv3 outputs decent maps but fails to capture any output from sample 3 and sample 7. Also, DeepLabv3 completely misses the smaller polyp in sample 6. Our proposed GAR-Net model outputs a refined segmentation map for all the samples. There is a small noise in the output map of sample 6 from our GAR-Net model. However, considering all the samples, our model outperforms other models with high efficacy, thus showing the superior performance of the proposed GAR-Net architecture on the Kvasir-SEG dataset also.

From Figure 7 and Figure 8, we can conclude that the proposed GAR-Net provides a more refined output map regardless of the noise or improper illuminations, providing a robust model for segmenting polyps from colonoscopy video frames.

#### 4.5.3. Strength of the Proposed Guided Attention Learning 

**Figure 9:**
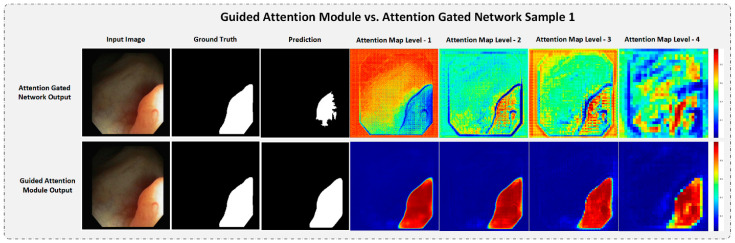
The attention visualization comparison: Attention Gated Network vs. Guided Attention Module with a test sample from CVC-ClinicDB dataset. Even in deeper layers, GAM is still able to capture refined attention maps, while Attention Gated Network only outputs coarse and broken attention maps.

**Figure 10:**
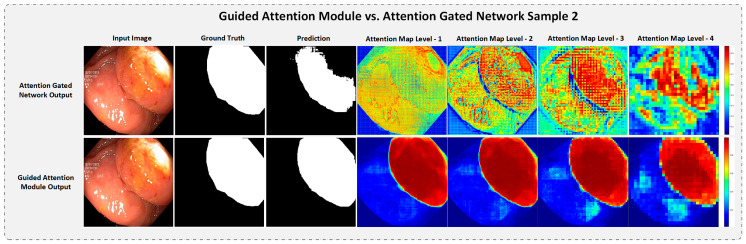
The attention visualization comparison compares Attention Gated Network vs. Guided Attention Module with a test sample from the Kvasir-SEG dataset. Notice even in deeper layers, GAM is still able to capture refined attention maps, while Attention Gated Network-only outputs coarse and broken attention maps.

To understand the significance of Guided Attention Learning in our GAR-Net architecture, we compared and visualized the attention map from the existing Attention Gated Mechanism [24] and the proposed GAM with GAL by taking a random test sample from both datasets. The attention visualization is presented in Figure 9 and Figure 10 for a random test sample in the CVC-ClinicDB dataset and Kvasir-SEG dataset, respectively.

From Figure 9, it is evident that the attention map of the Attention-Gated Network fails to capture the entire region of interest, hence, resulting in a broken, uneven output map. Moreover, as we go deeper into the network, the attention-gated network fails to provide a refined map and results in a very coarse attention map. However, the attention maps from our proposed GAM with GAL are highly refined even in the deeper layers of the network. Hence, we can get a more refined continuous output map.

From Figure 10, it is again evident that the attention-gated network completely fails to capture the region of interest at low-level layers (early stages of the network). However, in the deeper layers of the network, we can see that the attention-gated network results in a coarse feature map with a lot of noise, as it captures only some part of the output and lacks to obtain a refined attention map. But our proposed Guided Attention Learning successfully captures a refined attention map both in earlier and in deeper layers of the model. Hence, we can conclude that our Guided Attention Module with Guided Attention Learning outperforms the previously proposed Attention-gated network and boosts the model’s performance by capturing refined output maps. 

## 5. Conclusions

In this paper, we presented GAR-Net: Guided Attention-based Residual Network, which is an architecture designed to address the need for a more accurate and refined segmentation map for the colorectal polyps found in colonoscopy examinations. The proposed architecture takes advantage of residual blocks, and attention mechanisms to output refined segmentation maps. We have modified the residual block by including a convolution layer in the skip connection to suppress the noise and capture the refined low-level feature map. We have proposed a new attention mechanism that successfully captures a refined attention map both in earlier and in deeper layers of the model. The Guided Attention mechanism proposed for this GAR-Net architecture generates a more refined output map regardless of improper illuminations, providing a robust model for segmenting polyps from colonoscopy video frames. 

Comprehensive examinations and experiments were conducted using the benchmark CVC-ClinicDB dataset and Kvasir-SEG dataset to evaluate and assess the proposed model with the existing state-of-art architectures. Through experimental results, it is shown that our proposed GAR-Net model can provide a reliable and robust model with the highest Dice co-efficient and mIoU score, outperforming other proposed semantic segmentation models such as FCN8, U-Net, U-Net with Gated Attention, ResUNet, SegNet, and DeepLabv3. The computation overload is slightly high in our proposed GAR-Net architecture, as we used normal convolution over depth-wise separable convolution. We did an experiment with depth-wise separable convolution and found it quite detrimental, especially to the attention mechanisms. There is further research scope to improve this model by making it lightweight and incorporating spatial information in Guided Attention Learning. We can conclude that the proposed GAR-Net architecture can be considered a strong baseline for further investigation in the direction of developing a robust and clinically useful method for polyp segmentation from colonoscopy video frames.

## Figures and Tables

**Table 1:** The Results on the CVC-ClinicDB Dataset.

S No	Model	Dice Score	mIoU	Pixel Accuracy
**1**	**FCN8**	0.8724981	0.74308	0.9757281
**2**	**SegNet**	0.73168665	0.811956	0.97676927
**3**	**UNet**	0.8803434	0.767874	0.97531813
**4**	**UNet-Attn**	0.89131886	0.783651	0.97713906
**5**	**ResUNet**	0.89080334	0.781462	0.97680587
**6**	**DeepLabv3**	0.90001955	0.819477	0.9817561
**7**	**GAR-Net**	0.9100929	0.831234	0.9831491

**Table 2:** The Results for the Kvasir-SEG Dataset.

S No	Model	Dice Score	mIoU	Pixel Accuracy
**1**	**FCN8**	0.73635364	0.548514	0.9176181
**2**	**SegNet**	0.78634053	0.784731	0.9607266
**3**	**UNet**	0.85862905	0.750284	0.959373
**4**	**UNet-Attn**	0.8637741	0.754757	0.9587085
**5**	**ResUNet**	0.86858636	0.761282	0.9630264
**6**	**DeepLabv3**	0.87866044	0.805872	0.9693878
**7**	**GAR-Net**	0.8915458	0.815802	0.9717203

## Data Availability

The dataset used in this research is a benchmark dataset and is publicly available for researchers.
