# Correction: Raymann, J.; Rajalakshmi, R. GAR-Net: Guided Attention Residual Network for Polyp Segmentation from Colonoscopy Video Frames. *Diagnostics* 2023, *13*, 123

**DOI:** 10.3390/diagnostics14090900

**Published:** 2024-04-25

**Authors:** Joel Raymann, Ratnavel Rajalakshmi

**Affiliations:** 1 Faculty of Mathematics, University of Waterloo, Waterloo, ON N2L 3G1, Canada; 2 School of Computer Science and Engineering, Vellore Institute of Technology, Chennai 600127, India

There was an error in the original publication [1]. Section 2. Related Works contains paragraphs from the journal’s template.

A correction has been made by removing the last three paragraphs of Section 2. Related Works.

The authors state that the scientific conclusions are unaffected. This correction was approved by the Academic Editor. The original publication has also been updated.
